# ASO Author Reflections: Geographic Variation and Universal Challenges in the Patient Experience with Invasive Lobular Carcinoma

**DOI:** 10.1245/s10434-026-19644-8

**Published:** 2026-04-16

**Authors:** Claire E. Fanning, Jacqueline S. Jeruss

**Affiliations:** 1 University of Michigan Medical School, Ann Arbor, MI USA; 2 Department of Surgery and Rogel Cancer Center, University of Michigan, Ann Arbor, MI USA

## Past

Invasive lobular carcinoma (ILC) is the second most common breast cancer subtype, accounting for nearly 10–15% of all breast cancer diagnoses.^1^ Despite the prevalence of ILC, this subtype remains understudied when compared with infiltrating ductal cancer and presents unique diagnostic and management challenges. Owing to the distinctive growth pattern associated with ILC, which often produces subtle radiographic findings, ILC is more frequently multifocal, locally advanced, or metastatic at the time of diagnosis.^2^ These diagnostic complexities are further compounded by the relative underrepresentation of patients with ILC in clinical trials, which has limited the development of disease specific therapies.^3^ As a result, for both patients and providers, uncertainty surrounds the diagnosis and management of ILC. Radiologists, surgeons, pathologists, medical, and radiation oncologists face unique challenges when managing this disease, ranging from difficulties in accurately assessing tumor extent to interpreting intraoperative pathology findings and counseling patients regarding prognosis.^4^ While these clinical challenges can create significant stress for providers, the burden of uncertainty is ultimately endured by patients throughout the cancer care trajectory. Despite these challenges, minimal research has focused on the lived experience and psychological impact of ILC from the patient’s perspective. To this end, this study aimed to compare management and patient experiences with ILC between Europe and North America (NA) to identify factors that impacted the psychological well-being of patients with ILC and to provide insights to inform more transparent, empathetic, and patient-centered communication while navigating the formidable challenges of the ILC diagnosis.^5^

## Present

We examined the ILC patient experience including diagnosis, treatment, and follow-up care across 18 countries in NA and Europe implementing a survey study. While geographic differences were observed in stage at diagnosis and treatment recommendations, the study findings demonstrated that the emotional and psychological challenges associated with an ILC diagnosis were consistent across regions. European respondents presented with higher-stage disease and thus, were more likely to be treated with chemotherapy or antihormonal therapy, potentially reflecting differences in national screening guidelines and historical variances in breast cancer management between Europe and NA. However, despite these clinical differences, patients from both regions reported similar experiences of uncertainty, anxiety, and frustration related to their diagnosis, pervasive fear of delayed or missed diagnoses and recurrence, and difficulty obtaining clear information regarding the distinct behavior and management of the ILC subtype. These findings underscore that the challenges associated with ILC extend beyond diagnostic and therapeutic complexity. Patients frequently perceived gaps in medical communication, and mistrust and loss of confidence in healthcare providers. At the same time, many patients reported engaging in self-advocacy and connecting with support communities to better understand ILC management. Taken together, these results emphasize that while geographic differences influence aspects of ILC management, the psychological burden unique to this breast cancer subtype is universal and represents a critical, and often underrecognized, dimension of the impact of an ILC diagnosis.

## Future

Future efforts should focus on greater inclusion of patients with ILC in clinical trials in order to develop therapies that target the distinct biology of this disease. Improved and more consistent diagnostic approaches are also needed to help reduce the burden of both underestimation and overestimation of ILC in clinical imaging.^2^ Equally important is the need to address the communication and psychological challenges experienced by patients with ILC. Our findings demonstrate that uncertainty surrounding diagnosis and treatment can contribute to anxiety, mistrust, and harm the patient–provider relationship. Future efforts must prioritize improved communication between providers and patients regarding the unique clinical features of ILC and the unforeseen challenges that present during patient care. International qualitative studies that incorporate patient perspectives can help identify universal gaps in communication, education, and psychological support. By integrating such insights into clinical practice, the breast oncology community can work toward a more comprehensive model of care that addresses the uncertainty inherent to this diagnosis, while also prioritizing transparent, patient-centered communication to help minimize disease-related distress and improve patient-centered outcomes.
